# Greater public health impact of COVID-19 antigen detection tests

**DOI:** 10.1186/s12916-021-01956-z

**Published:** 2021-03-24

**Authors:** Akiko Kowada

**Affiliations:** Department of Occupational Health, Kitasato University Graduate School of Medical Sciences, 1-15-1 Kitasato, Minami-ku, Sagamihara, Kanagawa 252-0373 Japan

**Keywords:** COVID-19, SARS-CoV-2 diagnosis, Cost effectiveness, Public health

## Background

Early diagnosis of severe acute respiratory syndrome coronavirus 2 (SARS-CoV-2) infection prevents widespread transmission, manages outbreak control, improves poor prognosis, and reduces mortality of the Coronavirus disease 2019 (COVID-19). Policymakers, public health authorities, healthcare providers, and physicians have deployed COVID-19 control intervention and have made crucial decisions in choosing the appropriate COVID-19 diagnostic tests.

### Advantages of antigen detection tests

Antigen detection tests are diagnostic tests that quickly detect viral components or the virus directly without the need for a laboratory by testing samples collected from nasopharyngeal swabs and urine, and only reveal the active viral infection, not the recovery situation [1]. They are faster, cheaper, and easier-to-use compared with nucleic acid tests, and provide good clinical performance with more reliable results for patients with a shorter clinical course of the disease or a higher viral load [2]. The sensitivity of antigen detection tests are generally lower than that of nucleic acid tests, even though their specificity are comparable. Currently, a new COVID-19 antigen detection test with higher sensitivity (greater than 95%) and 100% specificity is available [3]. Antigen detection tests with high sensitivity and specificity are expected to play an increasingly important role in the early diagnosis of SARS-CoV-2 infection and will be more useful in hospitals, communities, and airports around the world.

### Impact of antigen detection tests

Ricks et al. now report in *BMC Medicine* greater healthcare cost-saving and health outcomes of antigen detection rapid diagnostic tests compared to nucleic acid tests for symptomatic persons [4]. The main outcomes in the study are the health system costs and health impacts (deaths averted and infectious days isolated). Antigen-detection rapid diagnostic test-led strategy is compared to a strategy based on nucleic acid tests and clinical judgment. Two important case scenarios are considered; in the hospital setting where the test is used to support infection control and treatment decisions among patients being admitted to hospital with respiratory symptoms, and in the community setting where the test is used in decentralized community clinics to identify cases of COVID-19 who should self-isolate. The authors extend their approach across countries by consulting experts from India, Nigeria, South Africa, and Brazil. They demonstrate that an antigen-detection rapid diagnostic test-led strategy is likely to improve health outcomes and be more inexpensive than a strategy based on nucleic acid tests and clinical judgment, in both hospital and community settings. Their approach is distinct from a conventional cost-effectiveness plane.

### Cost-effectiveness of public health strategies

The cost-effectiveness of SARS-CoV-2 diagnostic tests is a major global concern as well as the speed, accuracy, and simplicity of testing. Cost-effectiveness analysis facilitates evidence-based solutions for policymakers to prevent COVID-19 transmission. Silent transmission is also an important public health issue to control COVID-19. Approximately 50% of transmissions occur from asymptomatic or presymptomatic persons [5, 6]. In the future, an economic evaluation of public health strategies for patients and residents, including asymptomatic and presymptomatic persons with risk assessments, will become more critical. Public health strategies may be a combination of several effective preventive methods and diagnostic tests for high-risk groups and contacts [7, 8].

## Conclusions

Ricks et al.’s research strongly suggests that policymakers could choose COVID-19 antigen detection tests to control COVID-19 instead of nucleic acid tests for symptomatic persons, based on evidence, even with some limitations in the infectivity and transmission dynamics of SARS-CoV2 in the modelling analysis [4]. To make public health decisions that maximize health benefits for the largest number of patients and residents with limited resources, we need the continuous challenge to evaluate the cost-effectiveness and feasibility of novel SARS-CoV-2 diagnostic methods.

## Data Availability

Not applicable.
